# Predicting 90-day and long-term mortality in octogenarians undergoing radical cystectomy

**DOI:** 10.1186/s12894-018-0402-z

**Published:** 2018-10-22

**Authors:** Michael Froehner, Rainer Koch, Matthias Hübler, Ulrike Heberling, Vladimir Novotny, Stefan Zastrow, Oliver W. Hakenberg, Manfred P. Wirth

**Affiliations:** 1 Department of Urology, University Hospital Carl Gustav Carus, Technische Universität Dresden, Fetscherstrasse 74, D-01307 Dresden, Germany; 2 Department of Medical Statistics and Biometry, University Hospital Carl Gustav Carus, Technische Universität Dresden, Fetscherstrasse 74, D-01307 Dresden, Germany; 3 Department of Anesthesiology, University Hospital Carl Gustav Carus, Technische Universität Dresden, Fetscherstrasse 74, D-01307 Dresden, Germany; 4 Department of Urology, University of Rostock, Ernst-Heydemann-Strasse 6, D-18055 Rostock, Germany

**Keywords:** Bladder cancer, Radical cystectomy, 90-day mortality, Competing mortality, Comorbidity, Age

## Abstract

**Background:**

Radical cystectomy bears a considerable perioperative mortality risk particularly in elderly patients. In this study, we searched for predictors of perioperative and long-term competing (non-bladder cancer) mortality in elderly patients selected for radical cystectomy.

**Methods:**

We stratified 1184 consecutive patients who underwent radical cystectomy for high risk superficial or muscle-invasive urothelial or undifferentiated carcinoma of bladder into two groups (age < 80 years versus 80 years or older). Multivariable and cox proportional hazards models were used for data analysis.

**Results:**

Whereas Charlson score and the American Society of Anesthesiologists (ASA) physical status classification (but not age) were independent predictors of 90-day mortality in younger patients, only age predicted 90-day mortality in patients aged 80 years or older (odds ratio per year 1.24, *p* = 0.0422). Unlike in their younger counterparts, neither age nor Charlson score or ASA classification were predictors of long-term competing mortality in patients aged 80 years or older (hazard ratios 1.07-1.10, *p* values 0.21-0.77).

**Conclusions:**

This data suggest that extrapolations of perioperative mortality or long-term mortality risks of younger patients to octogenarians selected for radical cystectomy should be used with caution. Concerning 90-day mortality, chronological age provided prognostic information whereas comorbidity did not.

## Background

Radical cystectomy bears a considerable perioperative mortality risk particularly in elderly patients [1–5]. Robot-assisted surgery has been evaluated as a novel technique in order to decrease adverse outcome in elderly patients [6]. Currently, there is, however, still insufficient evidence to prefer any approach to radical cystectomy [7]. Elderly patients tend to be treated less aggressively although they may benefit from such treatment similarly as their younger counterparts [8, 9]. Since patients with a long remaining life expectancy and a low risk of perioperative mortality are more likely to benefit from radical surgery, identifying those patients could improve disease management. Until now, few tools are available in order to estimate the postoperative and long-term competing mortality risks in octogenarian candidates for radical cystectomy [1–5, 7–9].

## Methods

### Study sample

We studied a sample of 1184 consecutive patients who underwent radical cystectomy for high risk superficial or muscle-invasive urothelial or undifferentiated carcinoma of bladder at our institution between 1993 and 2015. Institutional review board approval was obtained (EK84032009). The patients were stratified into two groups by an a priori chosen cutoff (age < 80 years versus 80 years or older). Demographic data is given in Table 1.

**Table 1:** Demographic data of the study population in all patients, patients aged 80 years or older and patients younger than 80 years. Comorbidity profile and tumor-related parameters as well as 90-day mortality and 5-year bladder cancer specific and competing mortality rates were less favorable in patients aged 80 years or older compared with their younger counterparts

Parameter	Whole sample	< 80 years	80 years or older	*p*
Sample size	1184	1061	123	–
Mean follow-up (censored patients)	7.4 years	7.6 years	3.8 years	–
Median follow-up (censored patients)	6.2 years	6.5 years	2.5 years	–
Mean age	68.7 years	67.1 years	82.6 years	–
Bladder confined disease	684 (58%)	629 (59%)	55 (45%)	0.0020
Extravesical extension	500 (42%)	432 (41%)	68 (55%)	0.0020
Positive lymph nodes	308 (26%)	272 (26%)	36 (29%)	0.38
Extravesical extension or positive lymph nodes	576 (49%)	500 (47%)	76 (62%)	0.0021
Bladder confined disease and negative lymph nodes	608 (51%)	561 (53%)	47 (38%)	0.0021
ASA classes 3-4	493 (42%)	421 (40%)	72 (59%)	< 0.0001
Charlson score 2 or higher	449 (38%)	383 (36%)	66 (54%)	0.0001
Mean Charlson score	1.57	1.50	2.18	< 0.0001
Median Charlson score	1.00	1.00	2.00	–
CCS class 2 or higher	131 (11%)	109 (10%)	22 (18%)	0.0108
NYHA class 2 or higher	211 (18%)	177 (17%)	34 (28%)	0.0026
Female patients	255 (22%)	206 (19%)	49 (40%)	< 0.0001
Any neoadjuvant chemotherapy	55 (5%)	53 (5%)	2 (2%)	0.09
Adjuvant cisplatin-based chemotherapy	258 (22%)	257 (24%)	1 (1%)	< 0.0001
Current smokers	327 (28%)	315 (30%)	12 (10%)	< 0.0001
University degree/master craftsman	274 (23%)	250 (24%)	24 (20%)	0.31
Mean body mass index	27.0 kg/m^2^	27.0 kg/m^2^	26.7 kg/m^2^	0.26
Median body mass index	26.7 kg/m^2^	26.7 kg/m^2^	26.4 kg/m^2^	–
Continent diversion	390 (33%)	388 (37%)	2 (2%)	< 0.0001
Number of removed lymph nodes (if recorded)	18.4	18.8	15.2	< 0.0001
History of myocardial infarction	86 (7%)	72 (7%)	14 (11%)	0.06
Diabetes mellitus	288 (24%)	250 (24%)	38 (31%)	0.07
Lung disease	218 (18%)	196 (18%)	22 (18%)	0.87
Cerebrovascular disease	65 (5%)	56 (5%)	9 (7%)	0.35
Peripheral vascular disease	129 (11%)	111 (10%)	18 (15%)	0.16
Deaths from non-cancer causes	205	170	35	–
Deaths from bladder cancer	372	325	47	–
Deaths from second cancer	66	64	2	–
Deaths from unknown causes	7	5	2	–
90-day mortality	4.2%	3.7%	8.9%	< 0.0001
5-year bladder cancer-specific mortality	30.3%	28.9%	44.3%	0.0038
5-year competing (non-bladder cancer) mortality	14.1%	12.5%	28.7%	0.0005

*CCS* Classification of angina pectoris of the Canadian Cardiovascular Society [21]; *NYHA* Classification of cardiac insufficiency of the New York Heart Association [22]; *ASA* American Society Association physical status classification [11]

### Variables and data collection

Beside age as a continuous variable, numeric comorbidity (measured by the Charlson score [10]) and the clinical impression of the patient (measured by the American Society of Anesthesiologists (ASA) physical status classification [11]) (Table 2) and - concerning long-term competing (non-bladder cancer) mortality - a variety of single conditions were analyzed as possible predictors of outcome (Table 3). Comorbidity data was obtained from premedication records and discharge documents. Follow-up data were collected from urologists, general practitioners, the patients and their relatives, health insurance companies, local authorities and the local tumor register. All patients were observed for at least 90 days after surgery. Bladder cancer was considered the cause of death when uncontrolled disease progression was present at the time of death. Deaths from causes other than bladder cancer or unknown causes (*n* = 7) were considered deaths from competing causes. 90-day mortality (from all causes) and competing (non-bladder cancer) mortality were the study endpoints.

**Table 2:** A: Optimal multivariable logit models predicting 90-day mortality and B: Optimal multivariable proportional hazard models predicting non-bladder cancer (competing) mortality with 95% confidence intervals and *p* values in all patients, patients aged 80 years or older and patients younger than 80 years including the variables age, Charlson score and ASA classification

	Whole sample		< 80 years		80+ years	
A: Endpoint 90-day mortality						
Parameter	OR (95% CI)	*p*	OR (95% CI)	*p*	OR (95% CI)	*p*
Age (continuous variable, per year)	1.05 (1.01-1.09)	0.0106	n. s.*		1.24 (1.01-1.51)	0.0422
Charlson-Score (continuous variable, per point)	1.16 (1.02- 1.31)	0.0197	1.22 (1.07-1.39)	0.0029	n. s.**	
ASA classes 3-4 (versus classes 1-2)	6.95 (2.80-17.2)	< 0.0001	9.28 (3.11-27.8)	< 0.0001	n. s.**	
B: Endpoint non-bladder-cancer (competing) mortality						
Parameter	HR (95% CI)	*p*	HR (95% CI)	*p*	HR (95% CI)	*p*
Age (continuous variable, per year)	1.04 (1.02-1.05)	< 0.0001	1.04 (1.02-1.06)	< 0.0001	n. s.***	
Charlson-Score (continuous variable, per point)	1.17 (1.02-1.24)	< 0.0001	1.18 (1.11-1.26)	< 0.0001	n. s.***	
ASA classes 3-4 (versus classes 1-2)	1.59 (1.21-2.08)	0.0008	1.67 (1.25-2.25)	0.0006	n. s.***	

*ASA* American Society Association physical status classification [11]; *OR* Odds ratio; *HR* Hazard ratio; *CI* Confidence interval; *n. s*. Not significant. *Full model: age: OR 1.04 (0.99-1.09), p = 0.14, Charlson score: OR 1.21 (1.06-1.38), *p* = 0.0050, ASA classes 3-4: OR 8.48 (2.83-25.40), *p* = 0.0001. **Full model: age: OR 1.18 (0.97-1.48), *p* = 0.10, Charlson score: OR 0.90 (0.61-1.34), *p* = 0.60, ASA classes 3-4: OR 3.45 (0.66-17.95), *p* = 0.14. ***Full model: age: HR 1.08 (0.96-1.22), *p* = 0.21, Charlson score: HR 1.07 (0.96-1.27), *p* = 0.41, ASA classes 3-4: HR 1.10 (0.58-2.09), *p* = 0.77

**Table 3:** Optimal multivariable proportional hazard models with 95% confidence intervals and p values for competing risks predicting competing in all patients, patients aged 80 years or older and patients younger than 80 years, respectively investigating single conditions as possible predictors of competing mortality. Only single conditions occurring in at least 5 patients were included in the analysis

	Whole sample		< 80 years		80+ years	
Parameter	HR (95% CI)	*p*	HR (95% CI)	p	HR (95% CI)	*p*
Age (continuous variable, per year)	1.05 (1.03-1.06)	< 0.0001	1.06 (1.04-1.08)	< 0.0001		
Angina pectoris (CCS classes 2-4 versus 0-1)			1.89 (1.37-2.61)	0.0001		
Hypertension (versus none)						
History of thromboembolism (versus none)						
Myocardial infarction (versus none)	1.74 (1.21-2.51)	0.0029			2.20 (1.05-4.62)	0.0357
Cardiac insufficiency (NYHA classes 2-4 versus 0-1)						
Peripheral vascular disease (versus none)						
Cerebrovascular disease (versus none)						
Chronic lung disease (versus none)	1.41 (1.06-1.88)	0.0167				
Ulcer disease (versus none)						
Diabetes mellitus (versus none)	1.45 (1.12-1.88)	0.0051	1.37 (1.04-1.82)	0.0261		
Connective tissue disease (versus none)						
Hemiplegia (versus none)						
Moderate or severe renal disease (versus none)						
Solid tumor, leukemia or lymphoma (versus none)						
Liver disease (versus none)			2.38 (1.25-4.54)	0.0081		
Dementia (versus none)						
Current smoker (versus ex−/non-smokers^a^)	1.58 (1.21-2.07)	0.0008	1.75 (1.34-2.30)	< 0.0001		
Body mass index < 25 kg/m^2^ (versus 25+ kg/m^2^)						
ASA class 3-4 (versus classes 1-2)	1.68 (1.30-2.18)	< 0.0001	1.77 (1.34-2.34)	< 0.0001		
Male sex (versus female)			1.75 (1.18-2.62)	0.0052		

*CCS* Classification of angina pectoris of the Canadian Cardiovascular Society [21]; *NYHA* Classification of cardiac insufficiency of the New York Heart Association [22]; *ASA* American Society Association physical status classification [11]; *HR* Hazard ratio; *CI* Confidence interval; ^a^or unknown smoking status

### Statistical analyses

Concerning 90-day mortality, complete information for each patient (yes or no) was available. Multivariable logit models were used for the identification of predictors of 90-day mortality. Non-bladder cancer (competing) mortality was calculated from incomplete observations with censoring (and of observation in patients still alive) and competing (deaths from bladder cancer) events. Proportional hazard models for competing risks were used for the identification of predictors of non-bladder cancer (competing) mortality. Because of the limited number of events available for 90-day mortality, we dispensed from an analysis of multiple single conditions for this endpoint as done with long-term non-bladder cancer (competing) mortality (Table 3). The analyses were done with the Statistical Analysis Systems V9.4 statistical package (SAS Institute, Cary, NC).

## Results

**Fig. 1:**
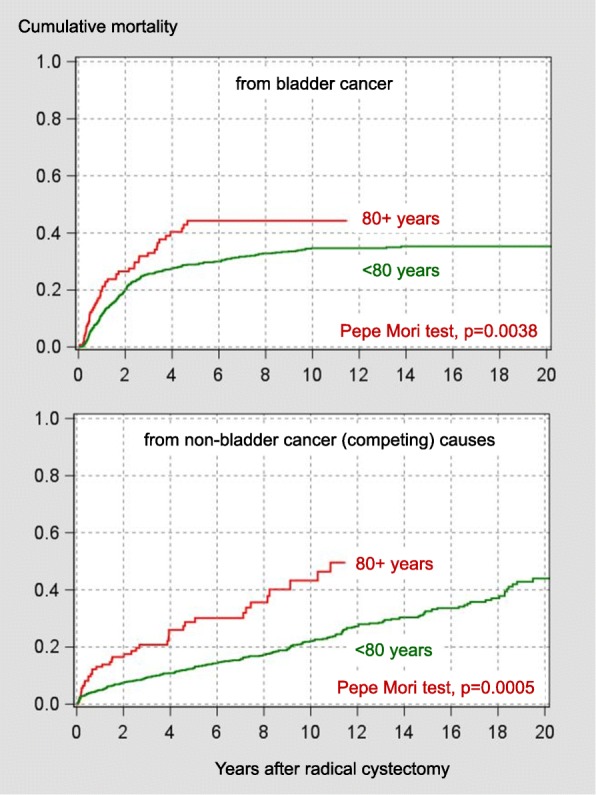
Cumulative mortality curves from bladder cancer and from causes other than bladder cancer (competing causes) stratified by the age cutoff of 80 years

Tumor-associated parameters (proportion of extravesical extension or positive lymph nodes), 5-year bladder cancer-specific mortality, 90-day mortality and non-bladder cancer (competing) mortality were less favorable in the octogenarian subgroup (Table 1). Octogenarians were more frequently female, less frequently current smokers, had a higher burden of comorbidity and did only rarely receive adjuvant and neoadjuvant chemotherapy (Table 1). Cumulative mortality curves from bladder cancer and from causes other than bladder cancer (competing causes) are shown in Fig. 1. Both types of mortality were higher in octogenarians compared with their younger counterparts (Fig. 1).

Whereas in younger patients the comorbidity measures Charlson score and ASA classification (but not age) were independent predictors of 90-day mortality, in those aged 80 years or older only chronological age was an independent predictor of 90-day mortality (Table 2). Remarkably, despite the range restriction of this variable in patients aged 80 years or older, chronological age became only an independent predictor of 90-day mortality after inclusion of this subgroup (Table 2).

**Fig. 2:**
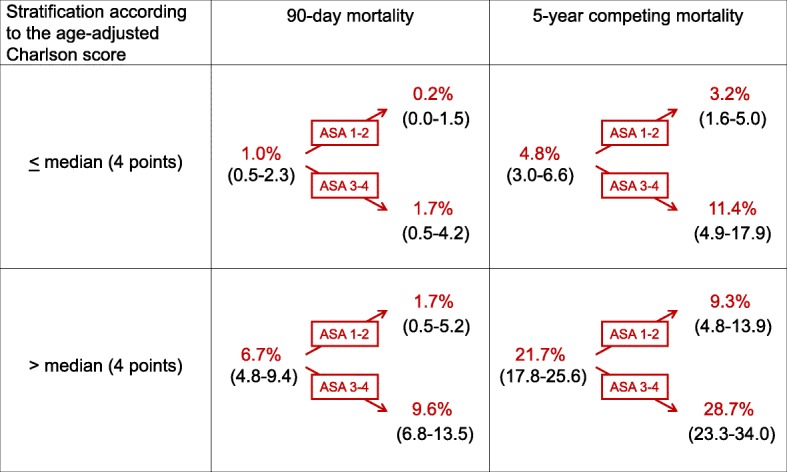
Impact of the stratification of patients younger than 80 years by the ASA classification on 90-day mortality and 5-year competing (non-bladder cancer) mortality rates after primary stratification by the age-adjusted Charlson score [16] (in brackets: 95% confidence intervals). Within the same risk group indicated by the age-adjusted Charlson score, the 90-day mortality differed by the factor 5-8 and 5-year competing mortality differed approximately by the factor 3 between patients with an ASA class 1-2 versus those with a ASA class 3-4. Such large differences are probably relevant for clinical decision making. For age-adjustment of the Charlson score, 1 point is added for an age of 50-59 years, 2 points for an age of 60-69 years, 3 points for an age of 70-79 years, 4 points for an age of 80-89 years and 5 points for an age of 90-99 years [16]

Whereas in younger patients age, Charlson score and ASA classification were independent predictors of long-term competing mortality with *p* values < 0.001, all three parameters were far apart from the significance level in patients aged 80 years or older (Table 2). In contrast, in younger patients, the ASA classification was of distinct and probably clinically meaningful impact on 90-day and long-term competing mortality after radical cystectomy in younger patients (Tables 2 and 3, Fig. 2). When single conditions were analyzed, in patients younger than 80 years a complex model containing age and six comorbidity-related variables predicted long-term competing mortality whereas in their older counterparts only one variable (history of myocardial infarction) was a significant predictor (Table 3).

## Discussion

This study suggests that extrapolations of 90-day mortality or long-term competing mortality risks of younger patients to octogenarians selected for radical cystectomy should be used with caution. Concerning 90-day mortality, in octogenarians selected for radical cystectomy chronological age could have greater impact than numeric comorbidity. In the full model predicting 90-day mortality in patients aged 80 years or older, the odds ratio of the Charlson score was lower than 1 indicating the loss of prognostic impact of numeric comorbidity (in contrast to findings in other types of cancer surgery [12]), whereas the ASA class with an odds ratio of 3.45 (95% confidence interval 0.66-17.95) sustained some of its prognostic impact visible in younger patients (Table 2). It is conceivable that with a larger sample size this classification might reach the significance level in the elderly subgroup as well.

Comorbidity has been found associated with perioperative death 90-day mortality and 5-year mortality after radical cystectomy [13, 14]. Nomograms have been developed and validated predicting all-cause mortality including variables related to age and comorbidity measured by the Charlson score [15].

In the current study, in patients younger than 80 years, particularly the ASA classification was of distinct and probably clinically meaningful impact on 90-day and long-term competing mortality after radical cystectomy in younger patients (Fig. 2). Whereas the 2017 guidelines of the American Urological Association (AUA) dispensed from detailed recommendations on comorbidity classifications [2], the current guidelines of the European Association of Urology (EAU) discouraged using the ASA classification as comorbidity measure in candidates for radical cystectomy [7]. The huge differences in 90-day and 5-year competing mortality observed after stratification by the ASA classification after previous stratification by the age-adjusted Charlson score [16] (the tool that is recommended by the EAU guidelines for comorbidity assessment [7]) in patients younger than 80 years suggest that the guideline’s discouragement of using the ASA classification should be revised for this subset of patients.

Compared with patients who were 70-79 years of age, octogenarians undergoing radical cystectomy had similar complication rates but increased mortality [17] underlining the need for an identification of vulnerable elderly patients prior to surgery. Furthermore, with a median of 23 months (95% confidence interval 20-27 months), in a recent large multicenter study, the overall survival rate has been reported to be relatively short octogenarians undergoing radical cystectomy [18]. In the current study, with a median of about 30 months, overall survival was something longer (Fig. 1). In a large octogenarian muscle-invasive bladder cancer sample including various types of management, in contrast to the current study comorbidity measured by the Charlson score was an independent predictor of overall mortality with a moderate association with mortality [18]. The inclusion of more impaired patients (only 26% underwent radical cystectomy [18]), an under-recording of less severe conditions in a multicenter cancer registry [18] and a larger sample size may be discussed as possible explanations for these differing findings.

It is conceivable that self-selection by accumulation of minor forms of chronic -diseases during the long life span and the elimination of severe life-threatening forms by premature mortality might diminish the prognostic significance of individual comorbid conditions in octogenarians. In geriatric patients undergoing emergency general surgery, in contrast to age and ASA classification frailty assessed by the Rockwood frailty index predicted postoperative and major complications [19]. Although it would be of interest, few data is available on the role of frailty assessment in elderly candidates for radical cystectomy until now [5].

This study has several limitations. The number of patients aged 80 years or older was limited. Concerning 90-day mortality, the number of events in this subgroup did not allow an analysis with a multitude of variables. Possibly, a multicenter approach including a huge number of octogenarians with well documented clinical data would be promising in order to identify factors other than age and comorbidity that could be associated with outcome in elderly candidates for radical cystectomy [20]. This study was focused on mortality; minor degree complications were not taken into consideration. 90-day mortality in octogenarians undergoing radical cystectomy is higher outside of academic centers [4]. It is possible that different results could be obtained outside an academic setting. Finally, it should be kept in mind that this analysis was based on patients selected for radical cystectomy; the results may not necessarily be extrapolated to a less strictly selected sample of elderly patients.

## Conclusions

This data suggest that extrapolations of perioperative mortality or long-term mortality risks of younger patients to octogenarians selected for radical cystectomy should be used with caution. Cystectomy should not be denied in octogenarians by numeric comorbidity. Concerning 90-day mortality, in octogenarians selected for radical cystectomy chronological age could have greater impact than numeric comorbidity.

## Abbreviations

ASA: American Society Association physical status classification
CCS: Classification of angina pectoris of the Canadian Cardiovascular Society
CI: confidence interval
HR: Hazard ratio
NYHA: Classification of cardiac insufficiency of the New York Heart Association
OR: Odds ratio
